# Reliability of Clinical Scoring Systems in the Diagnosis of Stroke Types in a Tertiary Care Center in South India

**DOI:** 10.7759/cureus.99182

**Published:** 2025-12-14

**Authors:** Namicharan Nabirajan, Sai Prasad Venkatramanan, Ashwath Magesh, Yogesh S, Hariharan C

**Affiliations:** 1 Internal Medicine, Madras Medical College and Rajiv Gandhi Government General Hospital, Chennai, IND

**Keywords:** allen stroke score, reliability, siriraj stroke score, south india, stroke scoring systems, validation

## Abstract

Introduction

Differentiation of stroke subtype is an essential first step in its management. In low-resource settings where access to neuroimaging is delayed, clinical scoring systems such as the Siriraj and Allen scores have been proposed as bedside tools to differentiate ischemic from hemorrhagic stroke. The primary objective of this study was to determine the diagnostic accuracy of the Siriraj and Allen scoring systems in differentiating ischemic and hemorrhagic stroke by comparison with computed tomography (CT) imaging.

Methods

A cross-sectional study was conducted among 156 patients admitted with acute stroke in a tertiary care center in South India. Clinical data were collected from patients within 24 hours of their admission to calculate the Siriraj Stroke Score and Allen Stroke Score. All patients underwent non-contrast CT brain imaging, interpreted by radiologists who were blinded to the scores. Sensitivity, specificity, and predictive values of both the scores for detecting hemorrhagic and ischemic stroke were calculated.

Results

Among the 156 patients (mean age: 54.1 years), 112 (71.8%) had ischemic stroke and 44 (28.2%) had hemorrhagic stroke. The Siriraj and Allen scores yielded equivocal results in 17.3% and 28.8% of cases, respectively. The Siriraj score demonstrated a sensitivity of 38.7% (95%CI, 23.8-56.2%) and a specificity of 96.9% (95%CI, 91.4-99.0%) for detecting hemorrhagic stroke. The corresponding positive and negative predictive values were 80.0% (95%CI, 54.8-92.9%) and 83.3% (95%CI, 75.5-89.1%), respectively. The Allen score showed a sensitivity of 35.7% (95%CI, 20.7-54.2%) and specificity of 88.0% (95%CI, 79.3-93.4%), with positive and negative predictive values of 50.0% (95%CI, 29.9-70.1%) and 80.2% (95%CI, 70.8-87.0%), respectively.

Conclusions

We found that the Siriraj and Allen stroke scores have poor diagnostic accuracy and yield a large proportion of equivocal results. We conclude that they cannot replace imaging in the differentiation of stroke subtype in the South Indian population.

## Introduction

Among the non-communicable diseases, stroke is the third leading cause of death and disability combined, with around seven million deaths and a global economic burden of 890 billion dollars attributed to it [1,2]. The bulk of the global stroke burden, 86.0% of deaths and 89.0% of disability adjusted life years (DALYs), originates in lower-income and lower-middle-income countries (LMICs) [3], such as India, where the annual incidence of acute stroke is estimated at 105-152 per lakh persons per year [4]. Of acute strokes in India, 68-80% are ischemic, with the remainder hemorrhagic in etiology [5].

An essential and urgent first step in the management of stroke is to rule out hemorrhage, i.e., to determine the subtype of stroke, as therapy like thrombolysis is contraindicated in hemorrhagic stroke and is very time sensitive. Neuroimaging, including non-contrast computed tomography (CT) and magnetic resonance imaging (MRI), is the gold standard for this crucial step, but access to timely imaging is an issue in the primary healthcare setting, compromising patient care [6,7]. 

Researchers have developed bedside scoring systems such as the Siriraj stroke score [8], Allen scoring system or Guy's Hospital score [9], Greek classification tool [10], and Besson score [11] to classify stroke subtypes using clinical and biochemical variables such as level of consciousness, blood pressure, white cell count, and focal signs. These scoring systems, developed based on observational studies done in their respective institutes, were made for application in the primary care setting in these regions.

Multiple studies have evaluated the performance of the scores in these regions as well as other regions and populations, with variable and inconsistent results [12-23]. In particular, there is limited evidence on their applicability in the South Indian setting, where clinical presentation patterns and comorbidities may differ. Earlier studies in this population are less robust because they involve smaller sample sizes and do not report statistical measures of significance [14-23].

This study evaluates and compares the diagnostic accuracy of Siriraj and Allen scores in identifying stroke subtype among patients at a tertiary care hospital in South India. We aimed to determine the reliability of the scores in the South Indian population in the settings where neuroimaging is delayed or unavailable.

## Materials and methods

The cross-sectional study was conducted on patients admitted with a clinical diagnosis of stroke to the Institute of Internal Medicine in Rajiv Gandhi Government General Hospital, Chennai, Tamil Nadu, India, over a period of three months from April to June 2024. The study was approved by the Institutional Ethics Committee of Madras Medical College (approval number: 56012024). Written informed consent was received from the patients after explaining, in their local language, the nature of the study and their freedom to refuse.

Study population

All acute stroke patients more than 18 years of age whose CT scan showed infarction or hemorrhagic supratentorial stroke and whose neurological deficit lasted for more than 24 hours were included in the study, provided they consented to participating in the study. Patients with stroke syndrome diagnosed to be due to underlying diseases that are not primarily vascular in nature, such as tumors, trauma, and tuberculosis; patients with subarachnoid hemorrhage; and those whose neurologic deficit resolved within 24 hours were excluded from the study.

Patients with a clinical diagnosis of stroke who satisfied the inclusion and exclusion criteria were recruited sequentially by consecutive sampling. A minimum required sample size of 117 was determined using Buderer’s formula [24], based on the expected sensitivity for hemorrhagic stroke.

Data collection

Data were collected from the patients within 24 hours of their admission by a combination of a brief, focused physical examination, lab reports, and a questionnaire to obtain specific socio-demographic and clinical parameters required to calculate the scores. Data were recorded using Google Forms and Google Sheets (Google LLC, Mountain View, California, United States). CT images of each patient were obtained and interpreted by experts at the Barnard Institute of Radiology, who were blinded from the scores and clinical details.

Clinical variables noted included level of consciousness, history of headache and vomiting within two hours of onset, diastolic blood pressure, presence of atheroma markers (angina, claudication, and diabetes [8]), apoplectic onset (defined as two or more of loss of consciousness, headache within two hours of stroke onset, vomiting within two hours of stroke onset, and neck stiffness), plantar response, history of hypertension, history of heart disease, and history of transient ischemic attack. In cases where the patient was unable to communicate, data was obtained from the patient’s attender. Whenever the history or physical sign was absent or ambiguous for a variable, it was assigned a score of zero, as mentioned in the protocol for calculating the scores in the original articles [8,9].

Data analysis

The data were used to calculate the Siriraj and Allen scores for each patient following the procedure and calculation suggested in their original articles (Table 1) [8,9]. The interpretation of each score was compared with the CT result of each patient and the sensitivity, specificity, and positive and negative predictive values of the scores in diagnosing hemorrhagic and ischemic stroke were calculated. The Wilson score method was used to calculate 95% CIs for sensitivity, specificity, positive predictive value, and negative predictive value. Analysis was done using Microsoft Excel (Microsoft Corporation, Redmond, Washington, United States).

**Table 1:** Stroke scores

Score	Number of variables	Formula	Interpretation
Allens score	8	Number of points = Apoplectic onset + Level of consciousness + Plantar responses + [Diastolic blood pressure (24 hours after admission) X 0.17] + Atheroma markers + History of hypertension+ Previous event (Transient ischaemic attack) + Heart disease + Constant (-12)	infarction: <4, hemorrhage: >24, equivocal: 4-24
Siriraj stroke score	5	Number of points = (2.5*level of consciousness) + (2*vomiting within 2 hours of onset) + (2*headache within 2 hours of onset) + (0.1 *diastolic blood pressure) -(3*atheroma markers) – 12 (constant)	hemorrhage: >1, infarction: < -1, equivocal: -1 to +1

## Results

A total of 156 stroke patients were evaluated, of which 112 were male and 44 were female. The mean age was 54.1 (range, 23-83). A total of 112 (71.8%) patients had a CT diagnosis of acute ischemic stroke, and 44 (28.2%) had acute hemorrhagic stroke. The demographic and clinical characteristics of the patients are displayed in Table 2.

**Table 2:** Clinical and demographic characteristics of the study population (N=156)

Characteristic	Type	Ischemic stroke (n=112)	Hemorrhagic stroke (n=44)
Sex, n (%)	male	76 (67.9%)	36 (81.8%)
	female	36 (32.1%)	8 (18.2%)
Age (years)	average	54.0	54.3
	range	23–86	31–80
Level of consciousness, n (%)	alert	89 (79.5%)	32 (72.7%)
	drowsy	23 (20.5%)	11 (25.0%)
	coma	0 (0%)	1 (2.3%)
Headache, n (%)	present	15 (13.4%)	17 (38.6%)
	absent	97 (86.6%)	27 (61.4%)
Vomiting, n (%)	present	11 (9.8%)	12 (27.3%)
	absent	101 (90.2%)	32 (72.7%)
Diastolic BP	average	89	100.0
	range	59–130	70–150
History of hypertension, n (%)	present	74 (66.1%)	31 (70.5%)
	absent	38 (33.9%)	13 (29.5%)
Atheroma markers, n (%)	present	59 (52.7%)	13 (29.5%)
	absent	53 (47.3%)	31 (70.5%)
History of transient ischemic attack, n (%)	present	17 (15.2%)	10 (22.7%)
	absent	95 (84.8%)	34 (77.2%)
Heart disease, n (%)	present	19 (17.0%)	3 (6.8%)
	absent	93 (83.0%)	41 (93.2%)
Plantar response, n (%)	normal	103 (92.0%)	41 (93.2%)
	bilateral extensor	9 (8.0%)	3 (6.8%)

**Figure 1:**
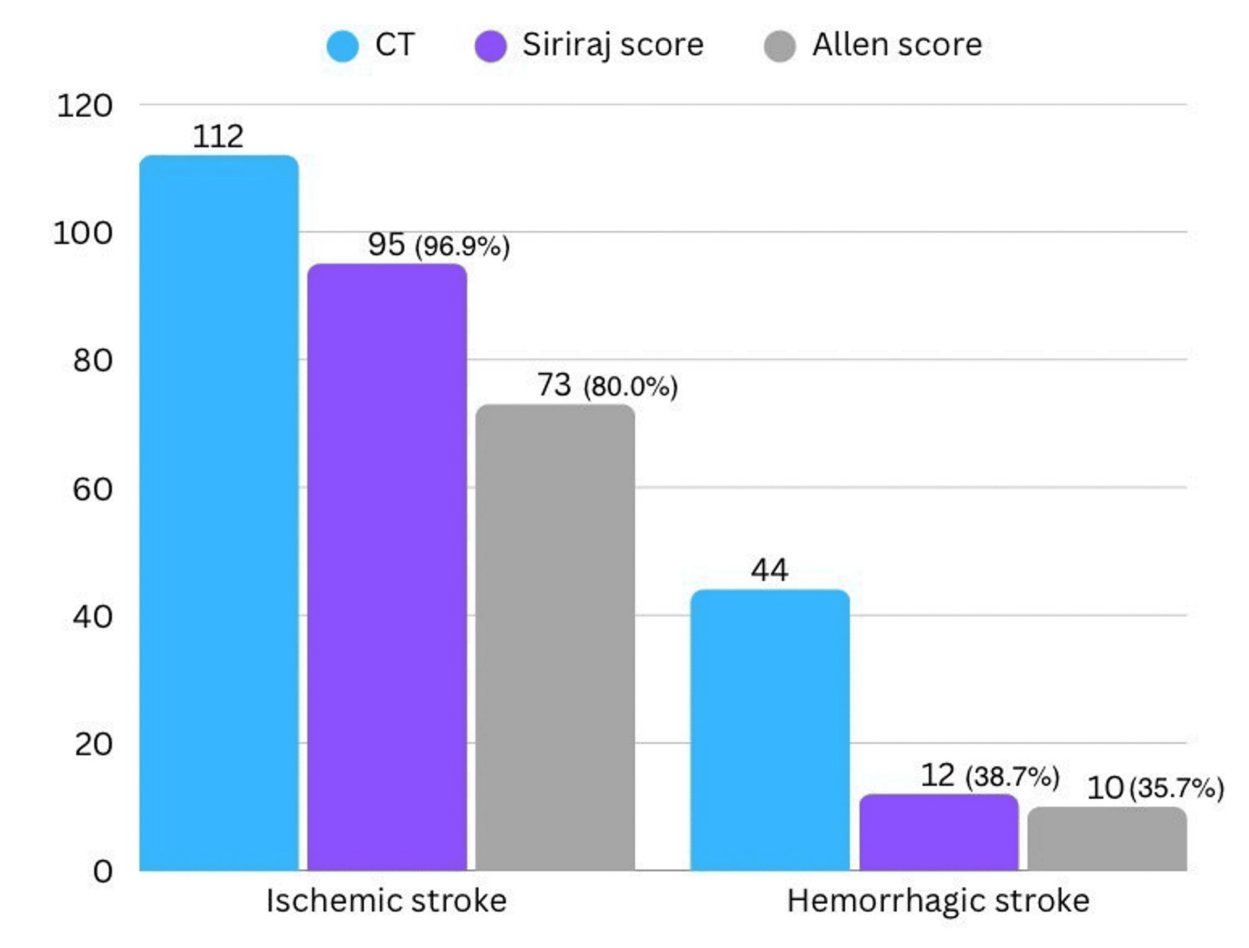
Comparison of CT, Siriraj and Allen scores in detecting Ischemic and hemorrhagic stroke

At admission, Siriraj scores and Allen scores were calculated, interpreted, and compared against CT results, as shown in Figure 1. The Siriraj score yielded a definitive prediction of stroke subtype in 129 out of the 156 cases (82.7%) and was interpreted as equivocal in the remaining. The Allen score was predictive of a stroke subtype in 111 (71.2%) cases, with equivocal results in 45 cases. Equivocal results were excluded from analysis.

The sensitivity, specificity, and positive and negative predictive values of the two scores in diagnosing hemorrhagic stroke and ischemic stroke were calculated from the data displayed in Table 3.

**Table 3:** Interpretation of Siriraj and Allen scores

Scores	Parameters	CT-diagnosed Hemorrhagic stroke (n=44)	CT-diagnosed Ischemic stroke (n=112)
Siriraj score interpretation	hemorrhagic stroke (>1)	12	3
	equivocal (-1 to +1)	13	14
	Ischemic stroke (< -1)	19	95
Allen score interpretation	hemorrhagic stroke (>24)	10	10
	equivocal (4 to 24)	16	29
	ischemic stroke (<4)	18	73

The Siriraj score demonstrated a sensitivity of 38.7% (95%CI, 23.8-56.2%) and a specificity of 96.9% (95%CI, 91.4-99.0%) for detecting hemorrhagic stroke. The corresponding positive and negative predictive values were 80.0% (95%CI, 54.8-92.9%) and 83.3% (95%CI, 75.5-89.1%), respectively. The Allen score showed a sensitivity of 35.7% (95%CI, 20.7-54.2%) and specificity of 88.0% (95%CI, 79.3-93.4%), with positive and negative predictive values of 50.0% (95%CI, 29.9-70.1%) and 80.2% (95%CI, 70.8-87.0%), respectively. These results are displayed in Table 4.

**Table 4:** Accuracy of the Siriraj and Allen scores in diagnosing stroke subtypes

Parameters	Hemorrhagic stroke		Ischemic stroke	
	Siriraj score	Allen score	Siriraj score	Allen score
Sensitivity	38.7%	35.7%	96.9%	88.0%
Specificity	96.9%	88.0%	38.7%	35.7%
Positive predictive value	80.0%	50%	83.3%	80.2%
Negative predictive value	83.3%	80.2%	80%	50%

## Discussion

The early differentiation of stroke into its pathophysiological subtype, ischemic or hemorrhagic, is essential; hemorrhage must be ruled out before the initiation of anticoagulants or antiplatelet therapy, a decision that must be made swiftly to optimize patient recovery. CT- and MRI-based methods are the gold standard, but in developing countries, access to sophisticated equipment is limited in the primary health care setting [6,7].

With this in mind, several bedside clinical scoring systems have been developed to rule out hemorrhagic stroke without relying on imaging. The first among these was made in 1984 in Guy’s Hospital in London by Allen et al. [9], followed by the Siriraj Hospital Medical School in Bangkok, Thailand, in 1991, which was proposed to be easier to calculate at the bedside [8]. Later, other scores, including the Greek score and Besson score, were developed [10,11].

Numerous studies over the last three decades have published varying results regarding the accuracy and utility of these scores, especially in demographics other than those of the scores' origins. There have been nine Indian studies, with four of these from South India, each primarily focusing on the Siriraj and Allen scores, with two studying the Greek score as well [14-23]. The sample sizes of these studies in South India were ≤100 and they have shown varying results, with sensitivities of these scores ranging from 50% to 90.90% in the diagnosis of hemorrhagic stroke (Table 5).

**Table 5:** Comparison of studies done in South India

Scores	Parameters	Current study (analysis excluding equivocal results) (N=156)	Rajan et al. [14] (analysis excluding equivocal results) (N=60)	Sreevani et al. [22] (analysis excluding equivocal results) (N=100)	Pavan et al. [23] (analysis excluding equivocal results) (N=100)	Ravi and Meghana [21] (N=60)
Siriraj score	Sensitivity	38.7%	50.0%	88.88%	77.27%	90.90%
	Specificity	96.9%	67.6%	87.5%	87.93%	86.49%
	Equivocal	17.3%	-	26%	20%	-
Allen score	Sensitivity	35.7%	-	88.23%	-	-
	Specificity	88.0%	-	92%	-	-
	Equivocal	28.8%	-	33%	-	-

Our study evaluated 156 patients with stroke admitted over three months at the Institute of Internal Medicine of the Rajiv Gandhi Government General Hospital in Chennai, Tamil Nadu. This hospital caters to the entire population of Tamil Nadu and sees patients from other states of South India, as well as referrals from the North. The scores were calculated using data collected from the patients at admission and compared with the CT diagnosis. 

A point to be noted is the number of equivocal results. The Siriraj and Allen scores were indecisive in classifying 17.3% and 28.8% of the cases, respectively. In the clinical setting, hemorrhage must be ruled out decisively and urgently for every case to administer life-saving interventions. So such a large percentage of indecisive cases questions the reliability of the scores as decision-making tools. These results also significantly lower the true overall sensitivity and specificity of the scores in detecting hemorrhage, as the equivocal cases were excluded from analysis.

For these scores developed to accurately rule out hemorrhage, the most important parameter is their sensitivity. The scores both performed poorly in this respect, with 38.7% and 35.7% for the Siriraj and Allen scores, respectively. Unfortunately, this means that a large percentage of hemorrhagic strokes would be falsely interpreted as ischemic if the scores are relied upon. The specificity was higher, meaning that a case characterized as hemorrhage by the scores correlated correctly with the CT 96.9% and 88.0% of the time for Siriraj and Allen scores, respectively. 

With a scoring system, a false negative would mean wrongly excluding intracranial hemorrhage and could lead to disastrous consequences following anticoagulation and antiplatelet therapy. Notably, the number of false negatives by the Siriraj score was 19 of 129, and the Allen score was 18 of 111, alarmingly high numbers. These results question the utility of the scores. The existence of such a significant number of false negatives and positives with these clinical scores points to the unpredictable variability in the clinical characteristics of strokes of both types, with each never following fixed rules in their presentation.

While our study data do support the fact that headache, vomiting, and loss of consciousness are seen more often in hemorrhagic than in ischemic stroke patients (Table 2), these and other simple clinical characteristics cannot be combined to reliably predict the subtype of stroke. There always exist outliers in clinical presentation, and even by raising the number of clinical parameters, as in the Allen score, the accuracy doesn’t increase, though the tediousness of applying the score does.

Limitations

The limitations of this study include the limited sample size of 156, as well as the fact that the study was conducted at a single center, which would affect the generalizability of its results. Additionally, the combination of both scores wasn’t studied; this could have potentially improved the overall sensitivity. The fact that the study was conducted in a tertiary care center brings in the possibility of severity and referral bias, as the cases that arrive at our hospital include a larger proportion of more complex cases referred from district hospitals. Some of these cases would have received medical interventions prior to arriving at our centre, and this could have altered the clinical variables, such as diastolic blood pressure. Some stroke signs and symptoms were reported by the patients' close family members, especially in cases where the patient was unresponsive or unable to communicate, which affects the reliability of the clinical data. Considering that several variables of each score, including apoplectic onset, history of chronic hypertension, angina, claudication, diabetes, previous transient ischemic attacks, and previous heart disease, would require patient history, relying on family members for this purpose (if previous medical records are unavailable) could lead to information bias. The absence of these data (including from patients' family members) would lead to these variables being assigned a value of zero, skewing the scores more in favor of ischemia. However, this is to be considered a limitation of the scoring systems rather than that of the study protocol itself, as in the setting where the scores would be used, the same disadvantages would still apply. In spite of these limitations, the study does offer insight into how these scores would perform if applied to the South Indian population.

## Conclusions

This study tested the performance of the Siriraj and Allen stroke subtype classification scores against CT imaging in a South Indian population. We found that a large percentage of strokes were interpreted as equivocal by both scores, and upon analysis of the remaining cases, the sensitivity of the scores in detecting hemorrhage was found to be poor, though the specificity was high. The exclusion of hemorrhage is a critical step in the management of stroke and requires a high level of accuracy, with unforgiving consequences if falsely interpreted and treated. We thus conclude that the Siriraj and Allen scores cannot replace imaging for this purpose in the South Indian population. However, with further modification and optimization of the scores to the clinical characteristics of the South Indian population, they could potentially be of diagnostic value. This should be explored in future studies.
